# Laboratory School Protocol Mini-Review: Use of Direct Observational and Objective Measures to Assess ADHD Treatment Response Across the Lifespan

**DOI:** 10.3389/fpsyg.2019.01796

**Published:** 2019-08-21

**Authors:** Sharon B. Wigal

**Affiliations:** AVIDA Inc., Newport Beach, CA, United States

**Keywords:** ADHD, laboratory, classroom, PERMP, SKAMP, children, preschoolers, adult workplace

## Abstract

**Introduction:**

The time of onset and the duration of treatment effect are important considerations in the choice of the medication to be prescribed in treating children, adolescents, and adults with ADHD. Early onset of effect may facilitate preparation for school, improved behavior during the trip to school, and attention during morning classes. Sustained treatment effect through afternoon and evening hours can be important because impairments associated with ADHD are not limited to the naturalistic classroom. Laboratory school protocols (LSPs) provide a simulated, rigorously controlled classroom setting environment and have proven valuable for providing pharmacokinetic and pharmacodynamic information about medications, and other treatments used in managing ADHD in school-aged children and across the lifespan.

**Methods:**

This paper is an invited mini-review of LSPs of stimulant medication, which includes data from multiple, randomized, double-blind, and placebo-controlled medication trials for ADHD. Assessment endpoints included the permanent product measure of performance (PERMP), Swanson, Kotkin, Agler, M-Flynn, and Pelham (SKAMP) rating scale in the preschool assessment laboratory (PAL), child/adolescent, and adult workplace environment (AWE) studies. These measures allow the study of improvement in attention and behavior in individuals with ADHD.

**Results:**

Analog classroom settings (LSP or AWE) have been used to assess immediate and modified-release stimulant formulations of medications to treat ADHD in multiple age groups. Results based on both subjective (e.g., SKAMP ratings) and objective (e.g., PERMP) measures are used as clinical outcomes in testing drugs currently in development for ADHD.

**Conclusion:**

The LSP and its extension to PAL and AWE settings continue to be used to assess the time-course of effect of ADHD medications because they provide valuable information in their respective structured, controlled environments.

## Introduction

The laboratory school protocol (LSP) and its associated methodology are important tools for measuring treatment onset, duration, and efficacy in individuals with ADHD. The LSP in its final form was developed by Drs. James Swanson, Sharon Wigal and colleagues in the early 1990’s at the University of California, Irvine (UCI). Much of this work in school-aged children has been defined and described elsewhere (Swanson et al., 2000, 2002) as highlighted in section “Tasks Typically Used in the LSP Highlighting Work in School-Aged Children” along with its history and revisions which include preschool aged children, adolescents, and adults (Wigal and Wigal, 2006).

Unlike some other classes of medications, weight-based dosing of stimulants has not proven effective, and age has failed to predict an effective dose (Newcorn et al., 2010). In addition, the large variations in the dose-response relationship (as many as fivefold) in children with ADHD (Markowitz and Patrick, 2008), underscore the value of using LSP studies to inform and predict stimulant response. The U.S. Food and Drug Administration (FDA) recently issued a guidance document regarding ADHD stimulant studies (U.S. Food and Drug Administration [FDA], 2019). They describe four primary efficacy measures that they consider acceptable for investigational new drug approval. Two of these measures are traditional symptom based rating scales: the ADHD Rating Scale and the Conners Rating Scale; the other two measures are the LSP measures known as the permanent product measure of performance (PERMP) math test and the Swanson, Kotkin, Agler, M-Flynn, and Pelham (SKAMP) rating scale (Swanson, 1992; Wigal et al., 1998; Wigal and Wigal, 2006). The symptom based rating scales are best suited for making a symptom-based diagnosis by an experienced clinician in a clinic setting of one patient at a time where time sensitivity is not critical beyond probing the past week or month. The LSP measures are best for assessing response to treatment by behaviorally trained observers in a group setting by measurement of functional behaviors in a time sensitive manner within and across a day.

The first ADHD treatment approval by the FDA developed with the LSP as described here was OROS^®^ Methylphenidate Hydrochloride (Concerta^®^; McNeil Consumer & Specialty Pharmaceuticals, Fort Washington, PA, United States) in August of 2000. The most recent U.S. FDA approvals were in August of 2018 for HLD200, a delayed-release and extended-release methylphenidate (DR/ER-MPH, JORNAY-PM^^TM^; Ironshore Pharmaceutical) (U.S. Food and Drug Administration [FDA], 2018), and in March 2019 for methylphenidate hydrochloride (Adhansia XR; Adlon Therapeutics, a Purdue Pharma subsidiary). The timing of drug administration is key to determining the time course of effects. Typical laboratory school studies utilized dosing schedules beginning in the morning, which allows for a pre-dose time point. Because the participants in the study would be on site for this measurement, a cohort of participants would be dosed simultaneously within a pre-scheduled dosing window. Depending on the specific clinical trial protocol, this window could be from 30 s to 15 min. Thus, recording the exact timing of dosing by direct observation in following the LSP provides a clear understanding of measured pharmacokinetic and/or pharmacodynamic effects with minimal variability of timing of measurements since dosing. More recent formulations involving evening dosing are typically dosed outside of the laboratory school site. The implication of this change in methodology is an introduction of uncertainty because the timing aspect of dose administration is determined by the parent(s) or individual patient without direct observation by study site personnel. These studies are more reliant on dosing reminder and adherence procedures. Different objective and subjective surrogate measures of efficacy can be obtained in the laboratory school setting. The key outcome measures developed specifically for the laboratory classroom are described in the following section along with some highlighted drug development data and the use of such measures across the lifespan.

## Tasks Typically Used in the LSP Highlighting Work in School-Aged Children

Treatment efficacy is generally similar amongst stimulants, but they differ by dosing, time course effects including duration of action and tolerability as measured by adverse events. Clinical efficacy is typically measured using the SKAMP Rating Scale (Swanson, 1992; Wigal et al., 1998; Wigal and Wigal, 2006), and the PERMP math test. A brief review of these measures is provided below.

The SKAMP rating scale was developed for a short rating period in a classroom setting rather than assessing long rating periods in home and/or clinical settings, and ratings may occur multiple times across the day during a schedule of structured classroom sessions followed by recess, meal, and playground activities. Various scoring methods have been used to categorize the SKAMP ratings representing attention and deportment as well as total score and work output, for example. Ratings are conducted on a seven-point scale of impairment in which 0 represents no impairment (normal) and 6 represents maximal impairment. Although perhaps obvious, it must be stated that these ratings reflect data collected *only* during the classroom probes and *do not include* any attention or behavior performance outside of the laboratory classroom during time spent with parents, counselors, coordinators, or site clinical research investigators. These raters must remain neutral and non-interactive with patients in any supportive or otherwise clinically meaningful ways during the laboratory classroom study days. In fact, their role as trained observers in the classroom precludes their involvement in any other form of study data collection. They are not permitted, by the LSP, to be involved in clinical ratings, phlebotomy, electrocardiography or blood pressure measurements, for example, in order to maintain their neutrality in data collection.

The study of stimulant responses using math tests in controlled settings traces back to Bradley (1937). As far as improvement in individual school subjects was concerned, the teachers were most impressed by changes in arithmetic performance, since speed of comprehension, and degree of accuracy and quantity of output were all favorably affected (Bradley, 1937, p. 582). The PERMP, a 10-min math test following a level-finding test, as described previously (Wigal and Wigal, 2006) is validated and time-sensitive to measure attention in subjects with ADHD. The PERMP measures the ability of a subject to initiate a task, self-monitor/stay on task and to complete written seatwork, and it does not test for mathematical ability or the ability to learn math. The difficulty of problems is adjusted to the pre-existing math skill level of each subject prior to treatment to ensure that each individual achieves high accuracy rates. The number of math problems attempted (PERMP-A) and the number of math problems answered correctly (PERMP-C) in a 10-min session are collected at multiple time points throughout a laboratory school study. In adults, the FDA has used the convention of calculating the PERMP Total Score (PERMP-T) as the sum of the PERMP-A and PERMP-C. The PERMP is a validated, time-sensitive, skill-adjusted, written math test designed to measure each individual’s ability to attend, initiate, and complete written seatwork, and it fulfills the following critical elements:

– A repetitive activity that the individual can perform with near 100% accuracy so that effort, not correctness, is the critical measurement.
– Yields a permanent product that is quantifiable.
– A variation in difficulty can be matched to the individual’s ability.
– A variation in the activity (e.g., different problems) minimizes learning or practice effects across days/weeks.
– An interest factor high enough for an individual to complete multiple times a day.
– A set of materials inexpensive enough so that multiple individuals can do the task at the same time.

The permanent product measure of performance assesses response to treatment, and it is related to at least 5 symptoms of ADHD (careless mistakes, fails to finish, easily distracted, can’t sustain attention, and avoids mentally effortful tasks). Unlike a continuous performance task (CPT), for example, which is only related to one ADHD symptom (i.e., can’t sustain attention), the PERMP has a much better chance of detecting response to treatment given the heterogeneity of symptoms in individual subjects with ADHD.

The defining characteristic of ADHD is not simply an attention deficit (total lack of attention), but rather inconsistent and misdirected attention. The expression of ADHD is usually contingent on context and task demands. Certain contexts or situations increase the likelihood of misdirected attention. Situations that are most likely to “bring out” ADHD symptoms are those that are boring, have delayed consequences (or infrequent feedback), are very familiar, include group settings, and have low salience. A defining characteristic of ADHD is that symptoms and impairments are variable throughout the day. ADHD may be described as a failure to manage the limited resource of attention depending on the individual and the context or demands on attention resources at any given time.

These laboratory school protocols measures are supposed to provide an index of “doing better” for patients, also referred to as response to treatment with the clinical goal of reduced impairment. Data analysis on each of these outcomes may examine average scores across the study day and/or statistical significance of treatment at specific time points when compared to placebo. The use of a randomized, double blind placebo control study design for inclusion of a placebo group across the study day is critically important for assessing such time course effects.

The initial laboratory school studies in elementary aged children described in Swanson et al. (2002) compared the effect of different delivery patterns of methylphenidate on SKAMP ratings and also depicted simulated pharmacokinetic profiles. This work illustrated the increased efficacy of an ascending drug delivery profile compared to other dosing regimens. Additionally, the use of highly trained observers in the classroom and rigorous protocol standardization *objectified* the subjective ratings and allowed for discrimination between treatment conditions.

**FIGURE 1:**
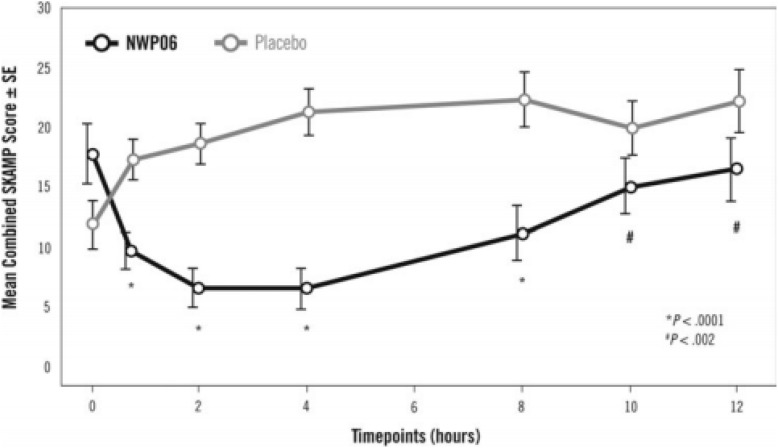
Laboratory Classroom SKAMP-Combined Scores. This figure is reprinted with the permission of the Journal of Child and Adolescent Psychopharmacology and publisher: Mary Ann Liebert Inc., New Rochelle, NY from Figure 2 in Wigal et al. (2013).

**FIGURE 2:**
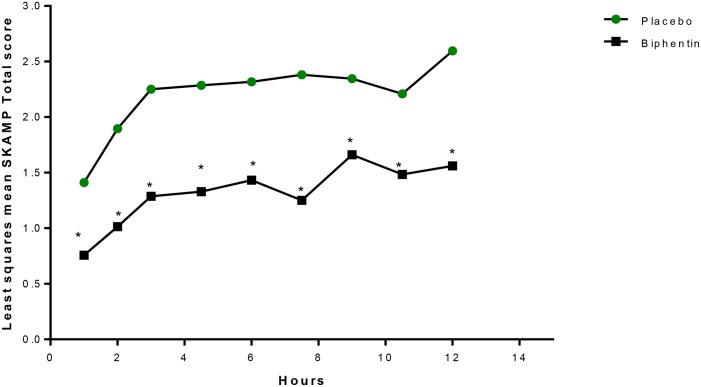
LS mean^+^ SKAMP-Total Scores Over Time (Evaluable Population, *n* = 20). LS, least-squares; mER, methylphenidate extended release; SKAMP, Swanson, Kotkin, Agler, M-Flynn, and Pelham. ^+^Mixed-effects analysis of covariance, with fixed terms for treatment, sequence, period, random term for subject within sequence, and covariate term for the predose value. ^∗^*P* ≤ 0.0261. This figure is reprinted with the permission of the Journal of Child and Adolescent Psychopharmacology and publisher: Mary Ann Liebert Inc., New Rochelle, NY from Figure 3 in Wigal et al. (2014).

Years later, another laboratory school study design feature was introduced in a different delivery form of methylphenidate, an extended release (ER) (Wigal et al., 2013) liquid formulation (Quillivant XR) compared to placebo in school aged children with ADHD. This study *a priori* selected a specific mean time point, 4 h, as the primary efficacy outcome measure to show significance of the active treatment on the SKAMP-Combined score rather than either averaging all time points or examining each time point of interest. In addition, the other time points tested, 0.75, 2, 8, 10, and 12 h each were significantly improved with treatment (see Figure 1). This type of statistical selection and ordering of time points influenced the design of more recent laboratory school studies. Similarly, another oral, non-liquid formulation, methylphenidate hydrochloride ER (Aptensio, 2015), has also demonstrated significant treatment vs. placebo effects on the SKAMP-Total score over time with time points from 1 hr through 12 h postdose as shown in Figure 2 (Wigal et al., 2014). One observation from these and other LSP data is that unlike clinician rating scales, both SKAMP and PERMP measures of the placebo group always move in the opposite direction from the medicated group. The SKAMP is more likely to show a small placebo response because it is a direct rating of observed behaviors with strict definitions. Objective measures like the PERMP typically show a small placebo response.

## Use of the Laboratory School Protocol in Preschool Children

It became evident at the time of the preschool ADHD treatment study (PATS) (see Greenhill et al., 2006; Kollins et al., 2006) that research in this understudied age group also could benefit from objective measurements in the laboratory classroom setting. The preschool version of this task does not use math problems at all. Instead it uses a comparable bead-stringing task as a tool and as another PERMP type of probe that is thought to be an analogous measure to the math test used in older age groups. The critical elements of a PERMP task as used in the preschool assessment laboratory (PAL) (see Wigal and Wigal, 2006, p. 96) also apply to this preschool task. However, additional use and replication of the bead-stringing task are warranted.

## Use of the Laboratory School Protocol in Adults

As the ADHD populations studied in clinical trials broadened, the LSP was adapted to allow for the evaluation of adult populations. The version of the LSP known as the adult workplace environment (AWE) was designed (Wigal and Wigal, 2006) to provoke ADHD-like behaviors by presenting challenging tasks, boring situations, and by mimicking workplace activities that require concentration, listening, organization, and mental effort. This study model also provides observational data, objective data, and some self-report data to help an expert clinician render a judgment about the presence of ADHD symptoms. To date, several studies have used the AWE to demonstrate treatment effects. A few such examples are provided below.

The AWE was first used in the study of adults with the triple bead formulation of Adderall treatment about 14 years ago, although these results were just recently published (Wigal et al., 2018). A chronologically later study though published earlier (Wigal et al., 2010) demonstrated a clear effect of lisdexamfetamine (LDX) compared to placebo in adults. More recently, the study of multi-layer release methylphenidate hydrochloride capsules following dose optimization in the clinic provided measurements in the AWE with time points from 1 to 16 h post-dose (Wigal et al., 2016). Each study has led to further refinement of AWE methodology including optimizing the number of PERMP math tests to reduce or eliminate the practice effect which is a nuisance variable in this age group and activities performed with adults during sessions outside of the classroom that will not confound classroom measurements while maintaining the contextual elements of the AWE. Because this test allows for multiple levels of difficulty, it works in the same predictable fashion in adults as in children once the correct level is assigned based on the pretest. However, because adults have less recent experience with performing math calculations by hand as compared to school-aged children, more practice sessions of the PERMP math test must be administered before the first data collection classroom.

## Discussion

The exemplar studies cited in this invited mini-review illustrate the adaptability of the LSP to shift the timing and dosing of treatment and its associated measures. Specifically, the objective measures of performance known as the PERMP, used in school-aged children, adolescents and adults, and bead stringing, used in preschoolers, allow measurement of treatment onset and offset effects. These measures are not sensitive to language based disabilities or cultural differences. The LSP also allows for continuity of measurement across the lifespan from preschool ages through older adults. Finally, the U.S. FDA has continued to request SKAMP and LSP study designs for drug development and timing of medication effects. In fact, there were only five clinical outcome assessments that were accepted by the FDA to determine if a drug treatment for ADHD has benefit. They are: the ADHD-RS, Conners, CGI-I, SKAMP, and PERMP (Pompilus et al., 2015). More recently, the FDA has drafted a guidance document to pharmaceutical industry that reduces acceptable clinical outcome measures for stimulant treatment approval to four measures, no longer including the CGI-I (U.S. Food and Drug Administration [FDA], 2019). The best way to collect PERMP outcome data in adults is in the context of the AWE. This is because the AWE provides the proper setting and thus, context, to ensure symptoms of ADHD will occur across a specified time period by provoking such conditions in a standardized manner.

Although external validity of the LSP can be inferred from the original Bradley (1937) work, the most important application is construct validity. The intent of AWE studies is to measure response to treatment. While it is true that children with ADHD typically do worse in school, and adults with ADHD typically do worse in the workplace, the results of AWE studies do not claim that treatment results in better grades or better vocational performance. The AWE was developed to include activities that require an attention and performance level needed in many workplace situations. In this way, symptoms of ADHD could be judged as improved (or not) during each cycle of activities. Thus, the LSP allows for a method to measure a reduction of ADHD symptoms that decrease at a given time point and continue throughout the day.

In summary, the LSP and AWE study designs provide FDA-approved methods to measure efficacy, onset, time response, and duration of effect of medication treatment. These outcomes are difficult to obtain in the clinic or naturalistic or a real-world setting. The LSP/AWE allows for structure in a controlled setting with rigorous objective and subjective measurements. These are the very features that may be seen as limitations (i.e., lower ecological or external validity) compared to naturalistic studies as described below. These LSP measures allow the study of improvement in seatwork compliance, attention, and deportment, for endpoint analyses of symptom change related to environment (i.e., home, school, work, social, and personal). The pairing of these two types of results from the laboratory classroom probes with more naturalistic clinical assessment outside of the laboratory classroom can help characterize the full picture of efficacy in the evaluation of new and existing drug treatments.

As mentioned, a key planned feature as well as limitation of the laboratory classroom design is the tight rigor that is incorporated in the scheduling of the study day. This coupled with the lengthier “school day” than is typical for any age group studied in the LSP and the inclusion of only subjects diagnosed with ADHD undermines the ecological validity of the data collected. Unfortunately, the study of healthy control subjects, either on stimulant treatment or without treatment for comparison in the LSP, have not been conducted nor published for reasons including ethical constraints. In addition, the novelty of the setting for participants, at least in initial test sessions, could reduce their response to treatment. While the LSP retains some aspects of the naturalistic setting such as a group setting, a boring task, structure that includes defined rules for expected behavior, and specific stop and start times, the measures used represent surrogates of real-world activities. The clinical value of data collected in any LSP study design is how well they guide community medical practitioners in naturalistic medication use and management of individual patients diagnosed with ADHD. Ultimately, the optimization of ADHD medication treatment in the clinic could be viewed as a naturalistic laboratory school study with *n* = 1.

## Author Contributions

SW conceived the mini-review, wrote and revised the manuscript, and read and approved the submitted version.

## Conflict of Interest Statement

SW was employed by AVIDA Inc., Newport Beach, CA, United States and has received research support from Akili Interactive Labs, Ironshore Pharmaceuticals & Development, Inc., Neurovance, Inc., NLS Pharma, NuTec, Otsuka, Pfizer, Purdue Pharma, Rho, Rhodes Pharmaceuticals, Shire, Sunovion Pharmaceuticals, Inc., and Tris Pharma, Inc. She has consulted for Arbor Pharmaceuticals, Atentiv, Ironshore Pharmaceuticals & Development, Inc., Neos Therapeutics, Neurovance, Inc., NLS Pharma, NuTec, Otsuka, Pfizer, Purdue Pharma, Rho, Rhodes Pharmaceuticals, Shire, Sunovion Pharmaceuticals, Inc., and Tris Pharma, Inc.; and served on speaker/advisory boards for Cingulate Therapeutics, Ironshore Pharmaceuticals & Development, Inc., NLS Pharma, NuTec, Otsuka, Pfizer, Purdue Pharma, Rho, Rhodes Pharmaceuticals, Shire, Sunovion Pharmaceuticals, Inc., Supernus Pharmaceuticals, Inc., TouchPoint Solutions, and Tris Pharma, Inc.
